# Evaluation of the Keros Classification of Olfactory Fossa by CT Scan in Qassim Region

**DOI:** 10.7759/cureus.22378

**Published:** 2022-02-19

**Authors:** Ziyad A Almushayti, Abdulhakeem N Almutairi, Mohammed A Almushayti, Haytham S Alzeadi, Emad A Alfadhel, Abdullah N AlSamani

**Affiliations:** 1 Department of Radiology, Qassim University, Buraydah, SAU; 2 Department of Otolaryngology, Head and Neck Surgery, Qassim University, Buraydah, SAU; 3 Department of Dentistry, Qassim University, Buraydah, SAU; 4 College of Medicine, Qassim University, Buraydah, SAU

**Keywords:** endoscopic sinus surgery, computed tomography, paranasal sinus, olfactory fossa, keros classification

## Abstract

Objective

Endoscopic sinus surgery (ESS) is now the most often utilized surgical procedure for treating chronic sinonasal disorders. Therefore, anatomical knowledge of its variations is required to avoid serious postoperative complications. Thus, careful preoperative examination for patients with a paranasal sinus CT scan is necessary. Our study aims to evaluate Keros types and their incidence by evaluating the olfactory fossa (OF) depth according to the Keros classification on paranasal sinus CT scans in the Qassim region.

Methods

A cross-sectional study was conducted between January 2018 and January 2021 on 148 patients with an average age of 32.59 ± 6.1 who had a non-enhanced paranasal sinus CT scan evaluated by a consultant radiologist using the PACS (picture archiving and communication system) software. Statistical analysis was performed using the statistical software package SPSS version 25 (IBM Corp., Armonk, NY). The chi-square test was used to analyze the relationship between findings and patient characteristics. Also, a p-value of < 0.05 was kept in mind to indicate statistical significance.

Results

The average depth of the right olfactory fossa (OF) was 5.1 mm with a standard deviation of 1.756 while it was 5.28 on the left side with a standard deviation of 1.66. According to the Keros classification, out of a total of 296 OF, type 1 was found in 84 (28.4%), type 2 in 188 (63.5%), and type 3 in 24 (8.1%). Consequently, the majority of cases were of type 2. Also, we found that type 2 was the most common on both sides in males, whereas, in females, type 2 was the most common on the left side and type 1 on the right side.

Conclusion

The study of the Keros classification is significantly important to evaluate the anatomy of the anterior skull base and give the surgeon knowledge about the depth of the olfactory fossa. Thus, a preoperative CT scan of the paranasal sinus is critical to ensure that the surgical approach is properly planned and possible surgical complications related to the anatomy of this area can be prevented. Our study showed that Keros type II is the most common, followed by type I and then type III.

## Introduction

In otolaryngology, chronic sinonasal diseases are one of the most common diseases requiring surgical attention [1]. Currently, endoscopic sinus surgery (ESS) is the most used surgical approach for treatment [2-3]. Therefore, to prevent serious postoperative complications, anatomical knowledge of its variations is mandatory. Thus, every patient should be examined carefully prior to the surgery [4].

The ethmoid bone is open superiorly; the roof is closed by the orbital plate of the frontal bone. Ethmoidal air cells indent this plate; each one is a fovea ethmoidalis. The thin, lateral cribriform lamella (one of the thinnest parts of the cranial base) forms the medial wall of the roof, extending from the middle turbinate to the cribriform plate, and the lateral wall of the olfactory fossa or niche. The olfactory fossa varies in depth and is frequently asymmetrical; it is at risk during sinus surgery [5].

The anterior ethmoidal artery may pass via the ethmoid sinus within the skull base, in the inferior surface of the skull base, or freely hang in a mesentery inferior to the skull base. Because the risk of damaging the artery during surgery is increased when it lies freely below the skull base, this relationship between the anterior ethmoidal artery and the skull base is critical [6].

The anterior ethmoidal artery is considered one of the anatomic features regarded as ‘‘high-risk’’ in endoscopic sinus surgery (ESS). It is especially dangerous because if damaged at its lateral end, it may lead to a retro-orbital hematoma and consequently may lead to optic nerve compression, resulting in blindness. In addition, a cerebrospinal fluid (CSF) leak or cerebrovascular insult or injury may result from a medial injury where the artery enters the lateral lamella of the cribriform plate [7].

In some sources, the olfactory fossa (OF) and the area around the ethmoid cellular are called the ‘danger zone’ [3-4,8]. Preoperative evaluation of the anterior cranial fossa, ethmoid roof, and adjacent bone structures to the olfactory fossa will lead to a safer route during surgery and decrease postoperative complications [9].

A paranasal sinus CT is a commonly used imaging method to evaluate the nasal cavity, paranasal sinuses, nasopharynx, and surrounding bone structures [9]. Cross-sectional imaging has been confirmed to be a useful tool for preoperative arrangement [10]. The best orientation for estimation of the relationship of the brain with the ethmoidal roof that associates closely with the surgical orientation is the coronal plane [11].

The Keros classification is widely used to evaluate the nasal roof depth [4]. As stated in the Keros classification, the depth of the olfactory fossa is estimated in three categories: type 1 (1-3 mm), type 2 (4-7 mm), and type 3 (8-16 mm) [12]. Type 3 is the most dangerous and important type of endoscopic sinus surgery and has a very thin cribriform plate [13]. The imbalance of the depth of both sides’ olfactory fossa or the height of the ethmoidal roof may lead to a greater risk of intracranial infiltration during endoscopic sinus surgery [3,14].

Our study aims to evaluate Keros types and their incidence by evaluating the OF depth according to the Keros classification on a paranasal sinus CT scan in the Qassim region. Besides the distribution of Keros types, age and gender differences were also evaluated.

## Materials and methods

This cross-sectional study was conducted at Qassim National Hospital, Buraydah, Qassim region, Saudi Arabia, between January 2018 and January 2021. The study included 148 patients (104 males and 44 females, with an average age of 32.59 ± 6.1) who underwent non-enhanced paranasal sinus CT scan evaluation having pathology or disease enough to obscure the regional anatomy. The evaluation was done by a consultant radiologist using the PACS (picture archiving and communication system) software.

The examinations were carried out on a 64-slice CT scanner (General Electronics, Boston, Massachusetts). Patients were kept in a supine position and data were acquired in an axial plane. Reconstruction was carried out in a coronal plane using a 3 mm slice thickness.

**Figure 1:**
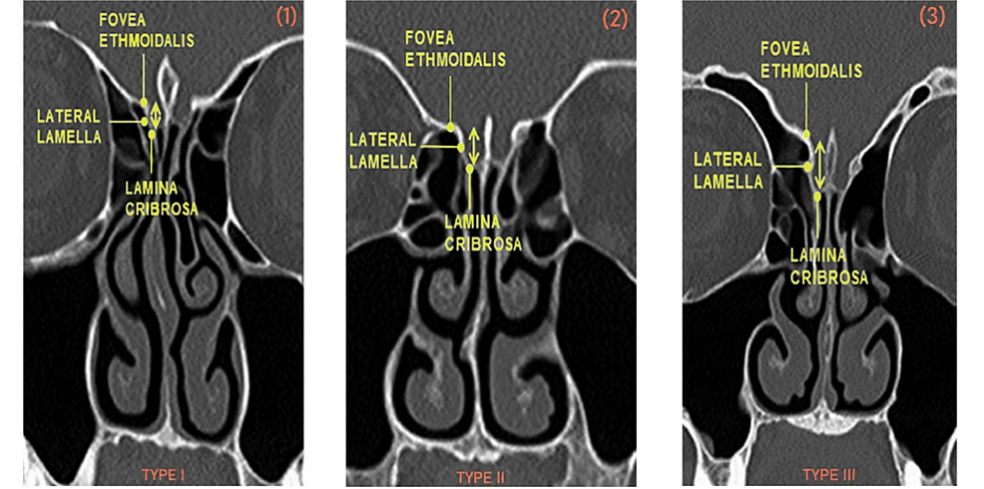
CT scans showing the Keros classification (1) Keros type I, (2) Keros type II, and (3) Keros type III

The depth of the olfactory fossa was measured and classified as per the Keros classification: type 1 (1-3 mm), type 2 (4-7 mm), and type 3 (8-16 mm) (Figure 1) [12].

In addition to patients who were below 18 years old, cases of trauma, post-nasal surgery, benign or malignant tumors of the sinonasal tract, and cases with asymmetrical morphology of the olfactory fossa were excluded from this study. Statistical analysis was performed using the statistical software package SPSS version 25 (IBM). The chi-square test was used to analyze the relationship between findings and patient characteristics. Also, a p-value < 0.05 was kept in mind to indicate statistical significance. In addition, oral and written informed consent was obtained from all participants. This project was approved under number 1443-810679 by the Regional Research Ethics Committee, Registered at National Committee of Bio & Med. Ethics (NCBE), Registration No. H-04-Q-001.

## Results

We included 148 valid cases with no missing cases in our study; 44 were females (29.73%) and 104 were males (70.27%). The average age of the patients was 32.59 ± 6.1. The oldest patient in males was 51-years-old and 37-years-old in females while the youngest was 24-year-old in males and 23-year-old in females.

The average depth of the right olfactory fossa was 5.1 mm with a standard deviation of 1.756 while the average depth of the left olfactory fossa was 5.28 with a standard deviation of 1.66 (Table 1).

**Table 1:** Comparison of the depth of the right and left olfactory fossae OF = olfactory fossa

	OF depth	
	Right side (MM)	Left side (MM)
N	148	148
Mean	5.1000	5.2865
Std. Deviation	1.75640	1.66020

According to the Keros classification, in the 296 olfactory fossae, type 1 was in 84 (28.4%), type 2 in 188 (63.5%), and type 3 in 24 (8.1%). Based on these data, the majority of cases, i.e., 188 (63.5%) were of type II (Table 2).

**Table 2:** Distribution of olfactory fossa according to the side and Keros classification

	Distribution of olfactory fossa according to the side and Keros classification					
	Right		Left		Total	
Keros type	N	%	N	%	N	%
Type I	52	35.1	32	21.6	84	28.4
Type II	88	59.5	100	67.6	188	63.5
Type III	8	5.4	16	10.8	24	8.1
Total	148	100	148	100	296	100

**Figure 2:**
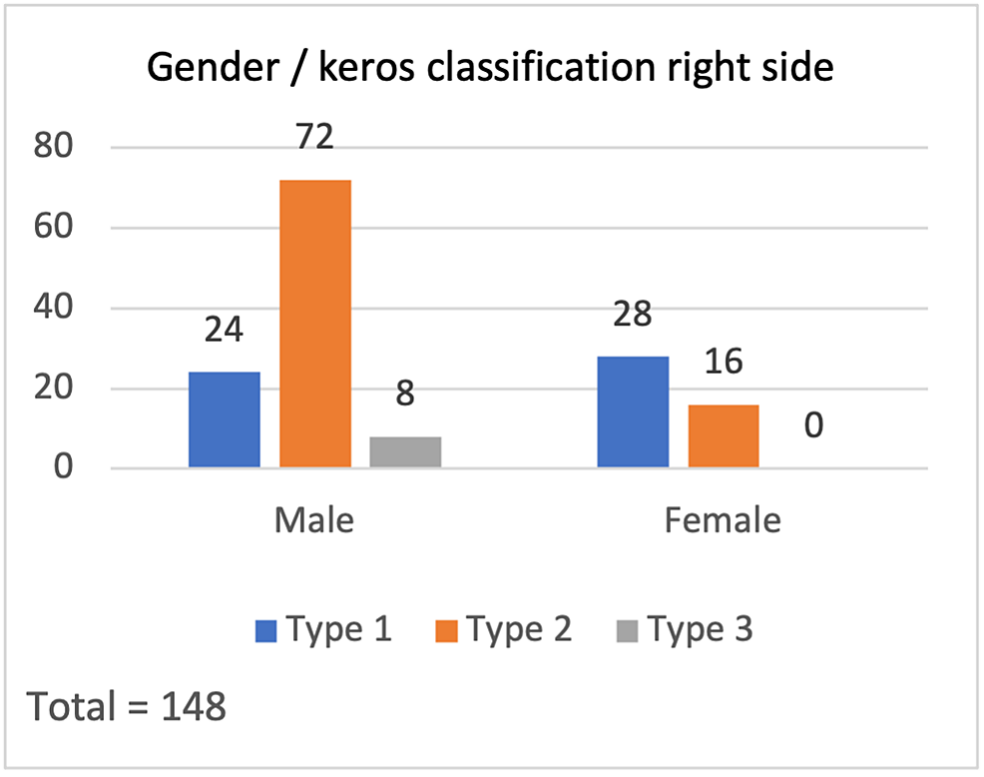
Gender / Keros classification - right side

**Figure 3:**
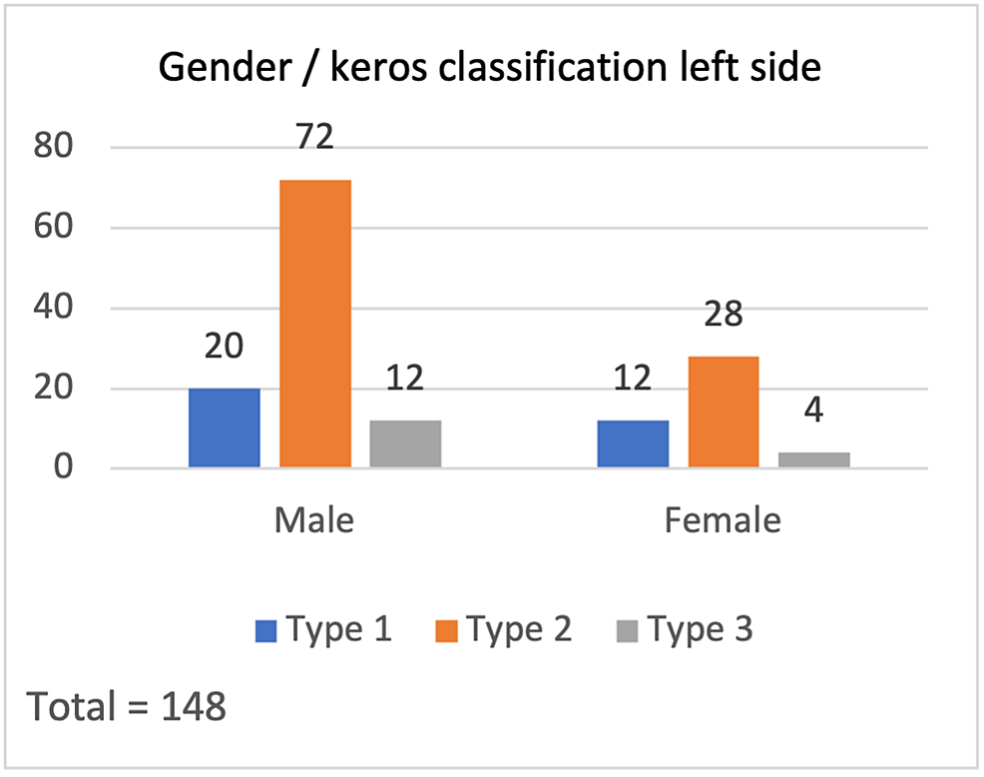
Gender / Keros classification - left side

According to the Keros classification, in the right-sided 148 olfactory fossae (Figure 2); Keros type I was present in 52 subjects (35.1%), type II in 88 (59.5%), and type III in eight (5.4%). For the total left-side olfactory fossae (Figure 3), Keros type I was present in 32 (21.6%) subjects, type II in 100 (67.6%), and type III in 16 (10.8%). According to these data, for males, type 2 is the most common on both sides while for females, type 2 was the most common on the left side and type 1 on the right side. In addition, there were significant differences between the Keros classification and the variables gender, right side (p-value = .002), and left side (p-value = .001).

## Discussion

After the development of the multi-slice technology, in addition to exposing the anatomical details in the best way, the thin-section paranasal sinus CT also provides an important aim for the diagnosis and treatment of paranasal sinus disease [15]. Coronal images can specifically be considered as maps in the assessment of the anatomy that is variable even between the two sides of the same person; there is a possibility of demonstrating areas at risk for complications in the arrangement of endoscopic nasal surgeries [1,16-17]. Although anatomical knowledge and experience decrease the probability of arising complications, ESS has been associated with some problematic symptoms because it is carried out in a complex region. The major complications are cerebrospinal fluid leakage, meningitis or intracranial vascular traumatization, and ocular traumatization; these complications are established in 0-1.5% of cases while the minor complications, including bleeding, infection, crusting, synechiae, ostial stenosis, and recurrence of the disease, occur in 1.1-20.8% of functional endoscopic sinus surgery cases [18-20]. Regarding the risk of complication development during ESS, the fovea ethmoidalis and lateral lamella are the most important parts of the skull base [3,21]. One of the oldest known studies on the olfactory fossa is the cadaveric study published by Keros P. in 1962, and the classification described here has been used based on many articles published up to date [12,22-23].

Our study observed Keros type II to be the most common, followed by type I and then type III. This is in agreement with studies conducted among the Saudi, Jordanian, Egyptian, Indian, Brazilian, and Turkish populations [24]. However, a study conducted in Khobar, Saudi Arabia reported type II as the most common presentation followed by type III then type I [25]. In contrast to our results, the studies performed among the Egyptian and Filipino populations demonstrated that the majority of studied subjects were classified as Keros type I, followed by type II and then type III [24].

Regarding gender, our data showed that Keros type 2 was the most observed on both sides of males. However, in females, type 2 was the most common on the left side, but on the right side, it was type 1. In the study conducted by Karatay et al. (2021), the mean OF depth on both sides was slightly lower than in our study [9]. When we compared the OF depth of both sides to the age and gender of all patients, we found a statistical significance (all are ≤ 0.05).

Keros type III is the most sensitive one, taking into account the major risk for iatrogenic lesion of the lateral lamella of the cribriform plate [26-27]. Asymmetry regarding the anterior of the skull base and the ethmoid roof is important for ESS. If the asymmetry exists, the rising of the ethmoid roof differs, and the fovea ethmoidalis of both sides may be at various levels. The side that has a low ethmoid roof will be more likely to have intracranial complications. The roof with low hanging may cause recurrent meningitis and cerebrospinal fluid fistula [28-29].

Factors that could be considered limitations were taking measurements by a single radiologist, all subjects in our study being adults (more than 18 years old), and the inclusion and exclusion criteria of the study could have been more thorough. A new hypothesis could be performed in a different study comparing the Keros classification in the pediatric and adult populations.

## Conclusions

The study of the Keros classification is significantly important to evaluate the anatomy of the anterior skull base and give the surgeon knowledge about the depth of the olfactory fossa. Consequently, we aim to approach a safe procedure during surgeries (especially functional endoscopic sinus surgery). Thus, a preoperative CT scan of the paranasal sinus is critical to ensure that the surgical approach is properly planned. Also, the regular use of the Keros classification for both sides in paranasal sinus CT reporting will aim to prevent surgical complications by providing important contributions to the surgical branches related to the anatomy of this area. In our study, Keros type II showed to be the commonest, followed by type I and then type III.
